# UV Treatment of Low-Temperature Processed SnO_2_ Electron Transport Layers for Planar Perovskite Solar Cells

**DOI:** 10.1186/s11671-018-2633-z

**Published:** 2018-07-20

**Authors:** Fumin Li, Mengqi Xu, Xingping Ma, Liang Shen, Liangxin Zhu, Yujuan Weng, Gentian Yue, Furui Tan, Chong Chen

**Affiliations:** 1 Henan Key Laboratory of Photovoltaic Materials, Henan University, 1 Jinming Road, Kaifeng, 475004 People’s Republic of China; 2 School of Physics and Electronics, Henan University, 1 Jinming Road, Kaifeng, 475004 People’s Republic of China; 3 State Key Laboratory on Integrated Optoelectronics, College of Electronic Science and Engineering, Jilin University, 2699 Qianjin Street, Changchun, 130012 People’s Republic of China

**Keywords:** UV treatment, Low-temperature, Tin oxide, Perovskite solar cells

## Abstract

We report a new method as UV treatment of low-temperature processed to obtain tin oxide (SnO_2_) electron transport layers (ETLs). The results show that the high quality of ETLs can be produced by controlling the thickness of the film while it is treated by UV. The thickness is dependent on the concentration of SnO_2_. Moreover, the conductivity and transmittance of the layer are dependent on the quality of the film. A planar perovskite solar cell is prepared based on this UV-treated film. The temperatures involved in the preparation process are less than 90 °C. An optimal power conversion efficiency of 14.36% is obtained at the concentration of SnO_2_ of 20%. This method of UV treatment SnO_2_ film at low temperature is suitable for the low-cost commercialized application.

## Background

Perovskite solar cells (PSCs) have attracted enormous research interest in recent years with power conversion efficiencies (PCE) enhancing from 3.8 to 22.1% [1–8]. In a typical perovskite solar cell either with or without mesoporous scaffold, an absorber layer is sandwiched between electrode-modified layers including the electron and hole transport layers (ETLs and HTLs, respectively), namely the mesoporous scaffold and planar hetero-junction architectures [9–11]. The high quality of the perovskite layer, which is smooth, compactive, and uniform, has a crucial impact on the device performance [12–14]. However, the quality of the bottom modified layer can directly affect the preparation of perovskite film. Typically, spin-coating method [15–17], hydrothermal synthesis method [18, 19], vacuum evaporation method [20], atomic layer deposition method [21], and electro-chemical deposition [22, 23] were adopted to improve the quality of the modified layers. And then, a compact modified layer was obtained by annealing and sintering at high temperature. The temperature is up to 450 and 180 °C when using TiO_2_ [24–27] and SnO_2_ [28–31] as the modified layer, respectively. The TiO_2_ was obtained by heat treatment of tetrabutyl titanate precursor, and the SnO_2_ was obtained by treatment of SnCl_2_ precursor [32]. However, the high temperature is not suitable for modern industrial manufacture.

To solve this problem, we present our preparation of compact layer by spin-coating SnO_2_ precursor and then treating by ultraviolet ozone (UVO). Here, tin oxide water solution is used as raw materials of SnO_2_. Moreover, the temperatures on each layer of the preparation of PSC are all at low temperature (less than 90 °C). It is easier to reduce technological difficulty of preparation process and to reduce production cost, which will be suitable for the industrial production. Our cells are based on CH_3_NH_3_PbI_3_ (MAPbI_3_), as a narrow band gap and high absorption material of visible light, which is processed by means of a one-step anti-solvent (OSAS) method [33–37]. The architecture of the planar hetero-junction PSC is Glass/ITO/SnO_2_/MAPbI_3_/Spiro-OMeTAD/Au. The MAPbI_3_ is sandwiched between SnO_2_ ETLs and Spiro-OMeTAD HTLs, respectively. After analyzing the surface morphology, surface element distribution, and light transmittance of the films, our results demonstrate that the SnO_2_-modified layer with compactness, purity, and high transmittance can be prepared by spin-coating and UVO treatment. Moreover, the high-performance planar PSCs were prepared at low temperature. The PCE of the PSC is 14.5% by optimizing the conditions of device preparation.

## Methods

### Materials and Precursor Preparation

Methylammonium iodide (MAI; Z99.5%) and lead iodide (PbI2; Z99.9%) were purchased from the Xi’an Polymer Light Technology Corp. Tin oxide (SnO_2_; 15% mass in H_2_O colloidal dispersion with a few organic solvents) was purchased from Alfa Aesar. 1,2-Dichlorobenzene (DCB; 99.5%) was purchased from J&K Scientific Ltd. *N*,*N*-Dimethylformamide (DMF; 99%), dimethylsulfoxide (DMSO; 99%), 2,2′, 7,7′-tetrakis(*N*,*N*-p-dimethoxyphenylamino)-9,9′-spirobifluorene (Spiro-OMeTAD), 4-tert-butylpyridine (TBP), and bis(trifluoromethylsulfonyl)-imide lithium salt (Li-TFSI) were purchased from Sigma Aldrich. Gold (Au; 99.995%) was purchased from China New Metal Materials Technology Co., Ltd. All the reagents were used without further purification.

### Fabrication of Devices

The PSC device has a structure of ITO/SnO_2_/MAPbI_3_/Spiro-OMeTAD/Au. The ITO glass plates (a sheet resistance of < 15 Ω/□) were pre-cleaned in an ultrasonic bath with acetone, ethanol, and de-ionized (DI) water for 15 min each, followed by drying with a nitrogen flow. Subsequently, the substrates were treated using ultraviolet ozone cleaner for 15 min at about 60 °C. The SnO_2_ thin films were prepared by spin-coating the SnO_2_ (*x* as 10, 15, 20, and 30%) precursor solution on the clean ITO glass substrates at 5000 rpm for 30 s and dried at 50 °C for 5 min, then treated by ultraviolet ozone cleaner for 60 min at about 60 °C. The solution concentrations of precursor were changed to 10, 15, 20, and 30% by diluting or condensing the original solution. A 1-M perovskite precursor of MAPbI_3_ was prepared by dissolving MAI and PbI_2_ in a 1:1 M ratio in 9:1 (*v*:*v*) mixed solvent of DMF and DMSO. Then, the precursors were stirred and heated at 50 °C overnight. For the active layer, the perovskite precursor was spin-coated at 4000 rpm. for 30 s on top of the SnO_2_ surface. Diethyl ether, as an anti-solvent agent, was drop-cast on the substrate at 5 s before the end of the spin. The samples were subsequently annealed at 90 °C for 10 min on hotplate in a glove-box and then cooled down for a few minutes. The typical thickness of MAPbI_3_ was about 300 nm. For HTM layer, 30 μL solution composing of 70 mM spiro-OMeTAD, 28.8 mM Li-TFSI, and 55 mM TBP in DCB was spin-coated on the perovskite layer at 5000 rpm. for 20 s. Finally, 100 nm of Au was thermally evaporated under high vacuum (5 × 10^−4^ Pa). The deposition rate which was monitored with a quartz oscillating thickness monitor (ULVAC, CRTM-9000) was approximately 5 Å/s. The active area of the device is 4 mm^2^.

### Characterization and Measurements

Current density–voltage (*J-V*) characteristics were measured using a computer-programmed Keithley 2400 source/meter under AM1.5G solar illumination using a Newport 94043A solar simulator. The intensity of the solar simulator was 100 mW/cm^2^. Light intensity was corrected by a standard silicon solar cell. The transmission spectrum was measured using ultraviolet/visible (UV–vis) spectrometer (Carry 5000). The surface morphology and structure of the as-prepared films were characterized using SEM (JSM-7001F, Japan Electron Optics Laboratory Co., Japan). The crystalline phase of as-prepared SnO_2_ film was confirmed by power X-ray diffractometry (XRD) (DX-2700, Dandong Fangyuan Instrument Co.Ltd., Dandong, China).

## Results and Discussion

**Fig. 1:**
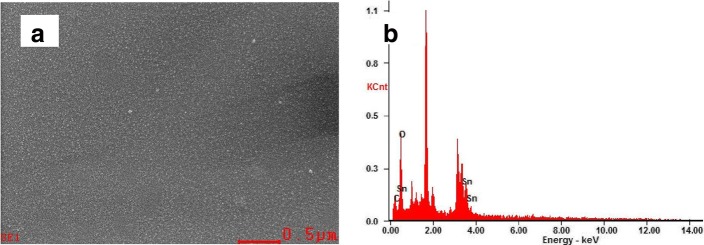
Surface SEM image of SnO_2_ (**a**) and the corresponding EDX spectra of ITO/SnO_2_ film

**Fig. 2:**
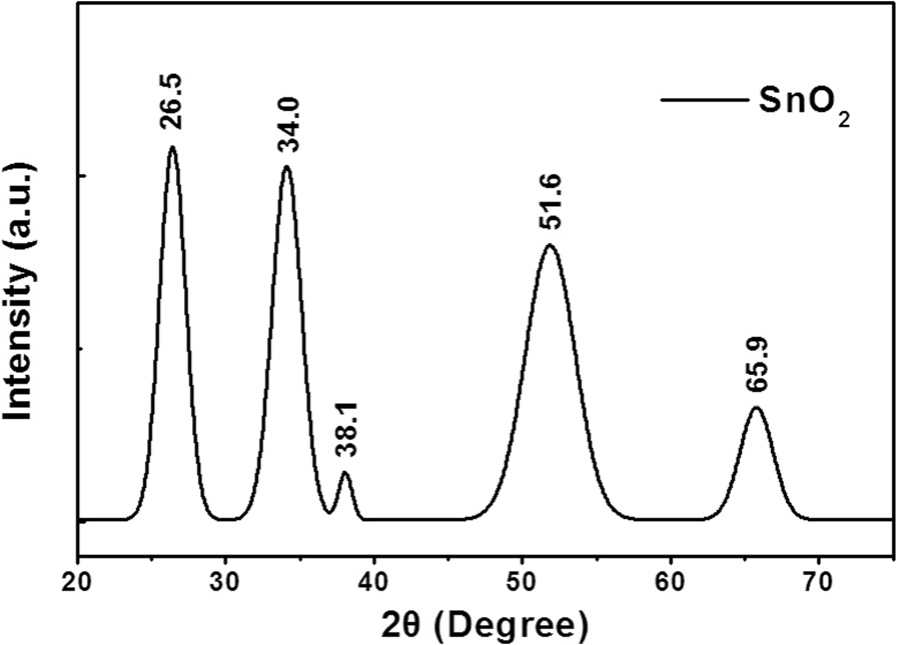
The X-ray diffraction (XRD) pattern of SnO_2_ after UV treatment

The UV/ozone can produce ultraviolet light that peaks nearly at 185 and 254 nm with photon energy of 647 and 472 kJ/mol, respectively, which are higher than the bond energy of C-C, C-O, and C-H of 346, 358, and 411 kJ/mol, respectively [38–40]. As a result, the UV light will easily break these chemical bonds while treating. In order to confirm it, SnO_2_ film with a concentration of 20% is selected for elemental distribution spectrometer (EDS) after UV treatment, and the distribution of the main components is investigated. Figure 1a shows the SEM of the selected film. The evenness and uniformity of the film are good at large scale at the bar of 0.5 um. Figure 1b shows the element distribution diagram, while the peak without mark is the peak position of the test electrode gold. As you can see, the Sn, O, and trace C element are included. Table 1 is the specific content of each element in the selected film. After UV treatment, the content of Sn and O in the film is greater than 99%, and the content of C is less than 1%. It can be recognized that most of the organic solvents are removed, and only Sn and O are left after UV treatment. So this way of processing can get the high purity SnO_2_ ETLs, which provides a possibility for the preparation of high-performance PSCs. Figure 2 shows the XRD pattern of SnO_2_ on slide glass after UV treatment. The XRD profile shows diffraction peaks at 2*θ* values of 26.5°, 34.0°, 38.1°, 51.6°, and 65.9°, which are identified as the reflections from (110), (101), (200), (211), and (301) planes of the rutile type tetragonal structure of SnO_2_ (JCPDS41-1445), respectively. The crystallite size of SnO_2_ was calculated using the Debye–Scherrer eq. (*D* = 0.89*λ*/*β*cos*θ*) [41], where *D* is mean crystallite size, *λ* is the X-ray wavelength, *θ* is the Bragg diffraction angle, and *β* is the peak width at half maximum. It provides an estimated crystallite size of 5.5 nm for the as-prepared sample.

**Table 1:** Specific content of each element

Element	Wt%	At%
CK	00.42	00.92
OK	49.29	87.82
SnL	50.29	11.26
Matrix	Correction	ZAF

**Fig. 3:**
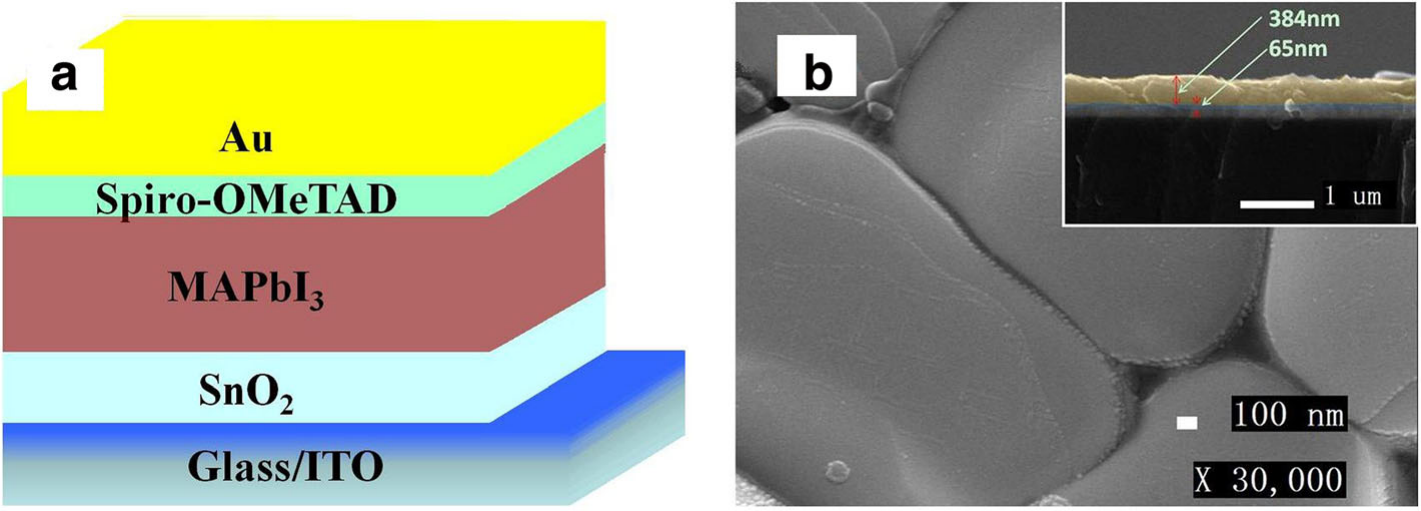
Structure diagram of the perovskite solar cell (**a**) and the SEM image of active layer (**b**)

Figure 3a is the structure diagram of the PSC. Figure 3b is the surface SEM image of the active layer, and the illustration is a cross-sectional view of the ITO/SnO_2_ (20%) /MAPbI_3_. It can be observed that the continuity of perovskite film is good. The particle size of the single perovskite crystal is larger than 1 μm; the transverse crystallization of the active layer is very good. The thickness of SnO_2_ (20%) is about 65 nm, and the thickness of perovskite is about 384 nm, which is expected to obtain high-performance perovskite solar cell.

**Fig. 4:**
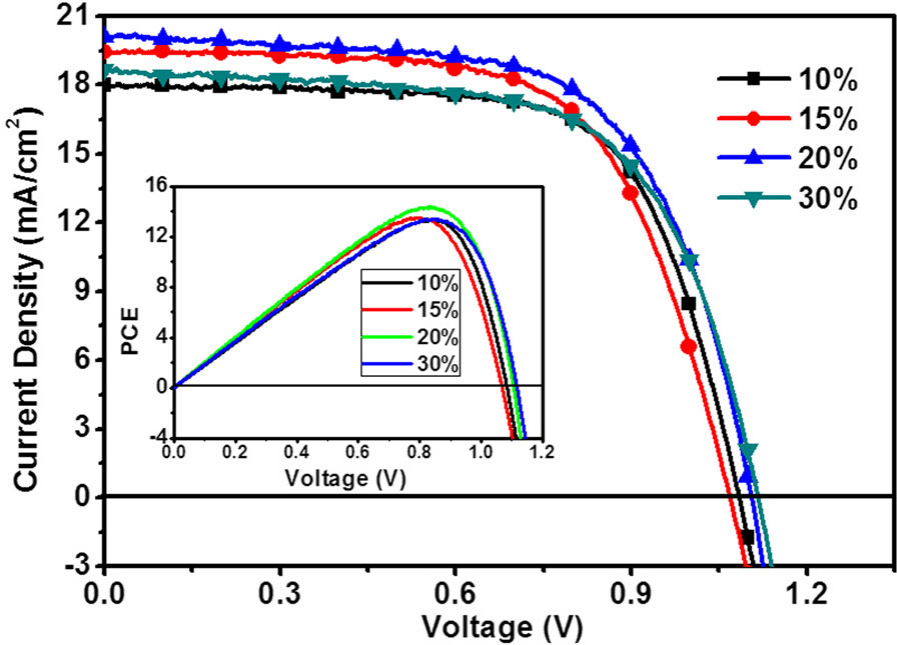
*J*-*V* characteristics of the device. The characteristics depend on the different concentrations of SnO_2_ which are varied from 10 to 30% under AM1.5G illumination of 100 mW/cm^2^. The inset shows the corresponding PCE-*V* curve

As shown in Fig. 4, the *J-V* characteristic curves of device ITO/SnO_2_(*x*)/MAPbI3/Spiro-OMeTAD/Au (*x* = 10, 15, 20, and 30%) under AM1.5G solar illumination of 100 mW/cm^2^ in ambient air. The detailed results are given in Table 2. It shows that *J*_sc_ of device increase first and then decrease with the increase of SnO_2_ concentration. *J*_sc_ of the device with 10% is the smallest and that with 20% is the largest. The probable reason is, when the concentration of SnO_2_ is changed, that the thickness of film increases which leads to increase resistance. Moreover, the light transmittance of film will be different due to the different thickness. *V*_oc_ of device increases with concentration of SnO_2_ increasing. The thick SnO_2_ film reduces the probability that the holes transport to the FTO electrode, which is easy to achieve for electrons. It is advantageous to reduce the recombination of carriers at the interface. When the concentration of SnO_2_ was 20%, the PSCs obtain an optimal performance with *J*_sc_ of 20.11 mA/cm^2^, *V*_oc_ of 1.11 V, FF of 0.643, PCE of 14.36%, Rs of 232.8 Ω, and Rsh of 15,868 Ω.

**Table 2:** Summary of PSC performance under illumination of 100 mW/cm^2^

Concentration	*V*_oc_ (V)	*J*_sc_ (mA/cm^2^)	PCE (%)	FF	Rs (Ω)	Rsh (Ω)
10%	1.08	17.92	13.32	0.688	265.4	42,011
15%	1.07	19.44	13.55	0.651	202.9	30,857
20%	1.11	20.11	14.36	0.643	182.8	15,868
30%	1.12	18.67	13.41	0.641	258.9	16,761

**Fig. 5:**
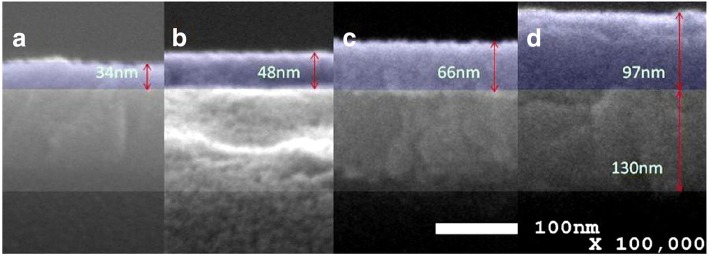
Cross-sectional SEM images of **a** the ITO/SnO_2_ (10%), **b** ITO/SnO_2_ (15%), **c** ITO/SnO_2_ (20%), and **d** ITO/SnO_2_ (30%)

**Fig. 6:**
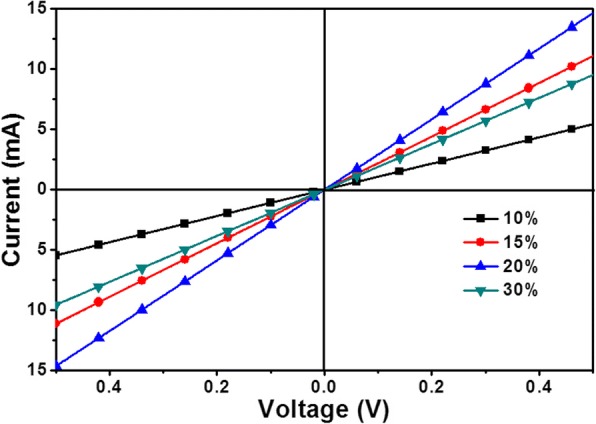
*I*-*V* curves of ITO/SnO_2_(*x*)/Au, *x* are 10, 15, 20, and 30%

Figure 5 shows the cross-sectional SEM images of SnO_2_ films. The image scale bar of the films is 100 nm, and its magnification is × 100,000. The thicknesses of the films which were prepared at different concentrations of SnO_2_ were 34 nm at 10%, 48 nm at 15%, 66 nm at 20%, and 97 nm at 30%, respectively. The thickness increased gradually by the increasing concentration of SnO_2_. In order to understand the influence on the vertical resistance of the thickness of SnO_2_ films, a resistance device was prepared with a structure as ITO/SnO_2_(*x*)/Au. Figure 6 shows the *I-V* curves. The resistance between ITO and Au were 98.6 Ω at 10%, 41.6 at 15%, 33.7 at 20%, and 50.8 at 30%. When the concentrations changed from 10 to 20%, the vertical resistance reduced, which increased when the concentration was up to 30%. It differs from the conventional knowledge that the resistance increases with the increase of thickness. To further analyze the reasons, the surface SEM of the films was investigated.

**Fig. 7:**
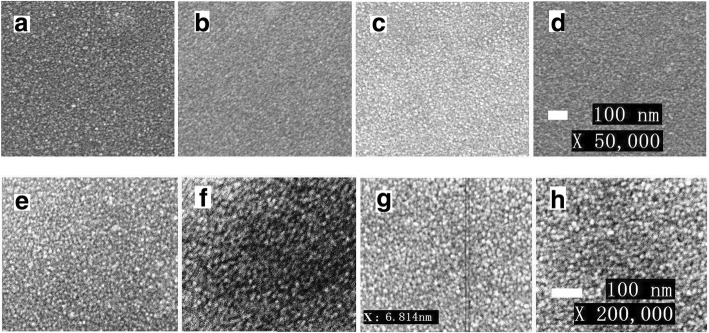
Top view SEM images of **a**–**d** the prepared ITO/SnO_2_(*x*) films at × 50,000 magnification, and **e**–**h** films at × 200,000 magnification

Figure 7a–d shows the top view SEM images of SnO_2_ films at × 50,000 magnification with a scale bar of 100 nm. And Fig. 7e–h shows the corresponding surface SEM images at × 200,000 magnification with a scale bar of 100 nm. It can be seen that the uniformity and smoothness of the films are very good at various concentrations, and the typical crystallite size of SnO_2_ is about 6.814 nm, which is quite approximate to that calculated of Debye–Scherrer eq. (5.5 nm), so that the high-quality active layer should be obtained when preparing the perovskite absorbance layer. There are just a few minor differences between them. This slight difference should be the reason that affects resistance. When the SnO_2_ concentration is 10%, the continuity of the films is poor, and some island groups appeared as shown in Fig. 7a, e. These defects on the surface introduce partial resistance value. The films are obviously uniform and even when the concentration increases to 20% as shown in Fig. 7b, c, f, g, which leads to an increase in electrical conductivity. While the concentration is up to 30%, the reunion situation is appeared which leads to an increase in the resistance. Moreover, the light transmittance of film was depended by the thickness of the modified layer, which affected the utilization of light by active materials.

**Fig. 8:**
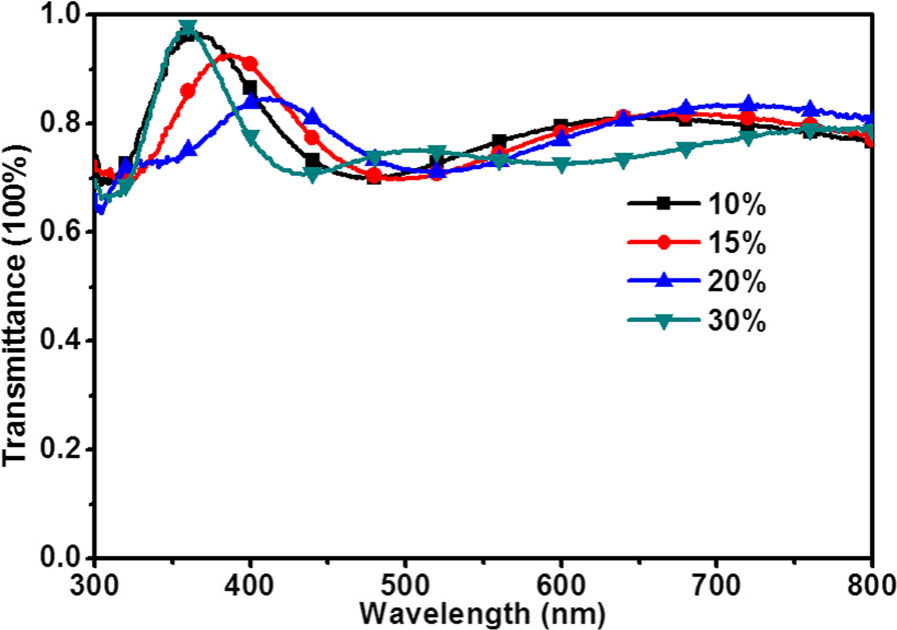
UV–vis transmission spectra of the ITO/SnO_2_(*x*) films

In order to understand the cause, we had tested the UV–vis transmission spectrum of the SnO_2_ (*x*) films, as shown in Fig. 8. It can be seen that the transmittance of the films exceeds 75% between 400 and 800 nm. The peaks are right on 616, 662, 718 nm, and more than 800 nm when the concentrations are 10, 15, 20, and 30%, respectively. With the increase of the thickness of SnO_2_, the transmission peak is red shifted. The absorption range of the MAPbI_3_ is between 300 and 760 nm. The transmitted lights are matched with that absorption range of perovskite while the concentrations are less than 20%. Therefore, the higher PCE could be obtained due to the more light utilization. When the concentration is 30%, the light absorption of active layer is attenuated that leads to a decrease in PCE. The utilization of light influences the performance of the PSCs. As a result, the PCE will be increased first and then decreased with the increase of concentration, which coincides with the previous results.

## Conclusions

In summary, we demonstrated a novel method as UVO treatment at low temperature which a high-quality SnO_2_ ETL could be prepared. High-performance PSCs were obtained by OSAS method. When the concentration of SnO_2_ was 20%, the PSCs obtained an optimal performance with PCE of 14.36%. The analysis results are shown that the conductivity and transmittance of the modified layer were depended on the thickness and uniformity of the film, and high-performance PSC could be obtained at suitable thickness of the modified film.

## Abbreviations

ETLs: Electron transport layers
FF: Fill factor
HTLs: Hole transport layers
*J*_sc_: Short-circuit photocurrent
OSAS: One-step anti-solvent
PCE: Power conversion efficiencies
PSCs: Perovskite solar cells
UVO: Ultraviolet ozone
*V*_oc_: Open-circuit voltage
